# Treatment Referrals Post-prohibition of Alcohol Exclusion Laws: Evidence from Colorado and Illinois

**DOI:** 10.1007/s11606-023-08544-2

**Published:** 2024-01-02

**Authors:** Sunday Azagba, Todd Ebling, Lingping Shan, Mark Hall, Mark Wolfson

**Affiliations:** 1 College of Nursing, Pennsylvania State University, University Park, PA USA; 2 Division of Biostatistics, College of Public Health, The Ohio State University, Columbus, OH USA; 3 Department of Social Science and Health Policy, Wake Forest School of Medicine, Winston-Salem, NC USA; 4 Department of Social Medicine, Population and Public Health, University of California Riverside School of Medicine, Riverside, CA USA

## Abstract

**Background:**

Individuals with alcohol-related disorders often encounter barriers to accessing treatment. One potential barrier is the state alcohol exclusion laws (AELs) that allow insurers to deny coverage for injuries or illnesses caused by alcohol intoxication. Several states have repealed AELs by prohibiting them completely, including banning exclusions in health and accident insurance policies, limiting their scope, or creating exemptions.

**Objectives:**

To examine whether prohibiting alcohol exclusions in health and accident insurance policies is associated with alcohol-related treatment admissions.

**Design:**

We used the 2002 to 2017 Treatment Episode Data Set and obtained data from several sources to control for state-level factors. We employed a heterogeneous difference-in-differences method and an event study to compare the treatment admissions in Colorado and Illinois, two states that uniquely repealed AELs, with control states that allowed or had no AELs.

**Main Measures:**

We used aggregated alcohol treatment admission for adults by healthcare referral: (i) with alcohol as the primary substance and (ii) with alcohol as the primary, secondary, or tertiary substance.

**Key Results:**

We found a significant relationship between AEL repeal and increased referrals. AEL repeal in Colorado and Illinois was associated with higher treatment admissions from 2008 to 2011 (average treatment effect on the treated: 2008 = 653, 2009 = 1161, 2010 = 1388, and 2011 = 2020). We also found that a longer duration of exposure to AEL repeal was associated with higher treatment admissions, but this effect faded after the fourth year post-treatment.

**Conclusions:**

Our study reveals a potential positive association between the repeal and prohibition of AELs and increased alcohol-related treatment admissions. These findings suggest that states could enhance treatment opportunities for alcohol-related disorders by reconsidering their stance on AELs. While our study highlights the possible public health benefits of repealing AELs, it also paves the way for additional studies in this domain.

**Supplementary Information:**

The online version contains supplementary material available at 10.1007/s11606-023-08544-2.

## INTRODUCTION

According to the World Health Organization, alcohol consumption was responsible for more than 3 million deaths worldwide in 2016, accounting for 5.3% of all deaths.^1^ In the USA, excessive alcohol use led to more than 140,000 deaths and 3.6 million years of potential life lost each year from 2015 to 2019.^2^ Excessive alcohol use (binge, heavy, and any drinking by pregnant women or people younger than age 21) can harm various aspects of health, both in the short and long term.^3^ Injuries, violence, alcohol poisoning, and risky sexual behaviors are among the immediate effects of excessive alcohol use.^4^ Some long-term effects of excessive alcohol use include mental health problems, liver disease, cancer, cardiovascular disease, and fetal alcohol spectrum disorders.^5,6^ Additionally, alcohol-related disorders continue to be a significant public health concern, contributing to global mortality and morbidity.^7^

Reducing alcohol’s negative impact on individuals and society is a critical public health challenge.^8^ One factor contributing to this challenge is that many people who suffer from alcohol-related disorders do not receive adequate treatment.^9^ According to the 2019 National Survey on Drug Use and Health, only about 6% of individuals aged 12 and older with alcohol-related disorders in the USA received treatment within the past year.^10^ Several barriers discourage people from seeking help for alcohol-related issues, including financial constraints, lack of access to quality care, and the pervasive role of alcohol in social and cultural contexts.^11–14^ Moreover, stigma is a significant factor that discourages people from acknowledging their alcohol issues and reaching out for support.^9,15,16^ While there is no universally accepted definition, Link and Phelan define stigma as “the co-occurrence of its components—labeling, stereotyping, separation, status loss, and discrimination—and further indicate that for stigmatization to occur, power must be exercised.”^17^ People with alcohol-related disorders often face stigma because they are stereotyped as irresponsible or morally flawed for their alcohol consumption.^18–20^ Policies that discriminate against or marginalize people who may have alcohol-related disorders can create a “structural stigma” that limits their opportunities, resources, and well-being, reinforcing the social stigma of alcohol use.^21^

Alcohol exclusion laws (AELs) are a barrier to accessing treatment for people with alcohol-related disorders.^22,23^ These laws allow health insurers to refuse coverage for injuries caused by intoxication. They were introduced in 1947 as part of the Uniform Accident and Sickness Policy Provision Law,^24^ a model law drafted by the National Association of Insurance Commissioners (NAIC). At that time, alcohol-related disorders were seen as a moral failing rather than a treatable disease^25^—and treatment options were limited.^26^ Subsequently, scientific evidence has shown that alcohol-related disorders are chronic and relapsing and require medical attention.^27^ AELs originally were based on a stigmatizing view of addiction,^28^ and they have yielded several unintended consequences. For example, data show that AELs do not save costs for insurers, as the amounts they pay for alcohol-related injuries continue to increase, having reached about $19 billion annually.^28^ Research has also demonstrated that screening and intervening for alcohol-related issues among injured patients can reduce alcohol consumption, hospital readmissions, and other adverse outcomes.^29–32^ Many patients are potentially denied this opportunity because of insurance exclusions allowed by AELs.^33^ Despite the NAIC’s 2001 recommendation to repeal or amend AELs due to their harmful effects, many states still have them in place. The number of states with AELs reached its highest point in 2000, with 40 states having such laws. Furthermore, the American Public Health Association has urged state legislatures and insurance commissioners to eliminate alcohol exclusions.^26^ This is due to the observed behavior of physicians in states where the UPPL is enforced, who often avoid measuring or documenting alcohol use. Such avoidance can lead to a lack of screening and intervention.^26^ In states where AELs persist, more individuals with alcohol-related disorders may go undiagnosed unnecessarily. If these individuals do not receive necessary screening and intervention due to insurance issues, repealing AELs could increase utilization and create more opportunities for treatment referrals.

More recently, numerous states have repealed their AELs; however, the effects of repeal have received only limited study. One prior study found that repealing these laws had no identifiable adverse effect on drinking behaviors.^34^ Another study found that AEL repeals increased alcohol treatment admissions from healthcare professional referrals, indicating a positive effect on access to care.^22^ Yet, not all states that have repealed their AELs have done so in the same way. Merely repealing a law that requires alcohol exclusions leaves the choice to insurers or possibly regulators as to whether to include them in the policies they issue to consumers. Going beyond a simple repeal, certain states also affirmatively banned alcohol exclusions in health and accident insurance policies. There is a lack of evidence on how different modes of AEL repeal across states may affect treatment outcomes.

The current study examines how a distinct version of repeal influenced alcohol treatment admissions, looking at two states. Among states that have repealed AELs, Colorado and Illinois are unique in that they not only repealed laws allowing alcohol exclusions but also prohibited exclusions in both health and accident insurance. Due to this distinctiveness, we hypothesized that this form of repeal was likely to have had observable associations with treatment admissions post-prohibition. To evaluate the effects of AEL repeal on alcohol-related treatment services, we compared the trends in alcohol-related treatment admissions in these two states with those of control groups with no similar policy. This approach allowed us to isolate the effects of state-specific dimensions of AEL repeal on treatment utilization while controlling for other factors that might influence alcohol-related treatment services. This study contributes to the literature on alcohol exclusion laws and public health in two ways. First, it explores how the dimensions of these laws affect health outcomes. Second, it applies a heterogeneous difference-in-differences (HDID) method that allows for varying treatment effects across both groups and time periods, rather than assuming a constant treatment effect as in the standard difference-in-differences (DID) approach.

## METHODS

### Data Sources

We obtained data on the utilization of treatment services for substance use disorders from the Treatment Episode Data Set (TEDS) from 2002 to 2017, excluding seven states with incomplete data. TEDS is a national administrative data system that monitors annual admissions and discharges to substance use disorder facilities that receive government funding.^35^ These facilities are mandated by the Substance Use Disorder and Mental Health Services Administration (SAMHSA) to report data on all clients, regardless of their health insurance status. State administrative systems collect the data and submit it to SAMHSA for processing. TEDS covers approximately 1.5 million substance use disorder treatment admissions annually, providing information on clients’ demographic characteristics, such as treatment service type and setting, employment status, and insurance status. Furthermore, TEDS records the three substances (primary, secondary, and tertiary) that prompted the treatment admission and the referral source (such as a doctor or healthcare provider, self, or criminal justice system) for each admission.

### Measures

#### Dependent Variables

We used the state identifiers in TEDS to calculate two state-level aggregate number of treatment admissions for patients aged 18 and over. We calculated (i) the number of admissions referred by healthcare professionals with alcohol as the primary substance and (ii) the number of admissions referred by healthcare professionals with alcohol as the primary, secondary, or tertiary substance use disorder diagnosis.

#### Independent Variables

We used the repeal of AELs as our main explanatory variable to examine its impact on alcohol-related treatment admissions. The repeal took effect on January 1, 2007, in Colorado and January 1, 2008, in Illinois. We also controlled for several state-level factors that could influence treatment admissions over time and across states, including economic conditions, demographic composition, alcohol taxation, and whether a state had Medicaid expansion in a particular year. We obtained data on these factors from various sources: the US Census Bureau provided data on insurance coverage rate (the percentage of people with any insurance) and state personal income per capita; the Bureau of Labor Statistics provided data on unemployment rate and median household income; the Tax Policy Center provided data on state beer taxes (adjusted for inflation to 2018 dollars); and the National Cancer Institute provided data on state population size, mean age, percentage male population, and percentage non-Hispanic white population. We used two data sets to estimate insurance coverage rates: the Current Population Survey Annual Social and Economic Supplement for 2002–2007 and the American Community Survey for years after 2007. These data sets have slightly different estimates but show a similar trend over time.^26^ We transformed state personal income per capita and population size into natural logarithms to reduce skewness. We chose control states based on their legal status regarding AEs (alcohol exclusions). We excluded states that explicitly banned AEs and included states that allowed AEs or had no AE law in the control group. We did not consider states that repealed AELs but did not prohibit AEs.

### Statistical Analysis

This study utilized a difference-in-differences approach to explore the relationship between AEL repeal and treatment admissions referrals by healthcare professionals. DID is a widely used quasi-experimental design for estimating the causal impacts of policy. It calculates the average effect of the treatment on the treated group (those exposed to a policy or intervention of interest) by comparing the difference in outcomes between the treated and control groups before and after the treatment.^36^ A standard DID with two groups (one treatment and one control group) and two time periods (one period before and one after policy or intervention) takes the following form:

1 $$\delta =E\left({Y}_{2t}\right)-E\left({Y}_{2c}\right)-E\left({Y}_{1t}\right)-E\left({Y}_{1c}\right)$$

where *δ* is the DID effect and the observed outcomes for the treatment group and the control group after the policy are represented as *Y*_2*t*_ and *Y*_2*c*_, respectively. Similarly, *Y*_1*t*_ and *Y*_1*c*_ are the observed outcomes for both the treatment and control groups before the policy. A regression version of the DID approach that adjusts for covariates can be represented as

2 $${Y}_{it}={\alpha}_{i}+{\lambda}_{t}+\delta {D}_{it}+{\textrm{X}}_{\textrm{it}}^{\prime }\ \beta +{\epsilon}_{it}$$

where *Y*_*it*_ denotes the outcome of interest for unit *i* (in our case, state) at time *t*. The equation includes, *α*_*i*_, a fixed effect for unit, and *λ*_*t*_, a time-fixed effect. *D*_*it*_ is an indicator of the treatment (policy) status, X_it_ is a vector of covariates, and *ϵ*_*it*_ is an errors term. The parameter, *δ*, captures the average effect of the treatment on the treated units. However, in settings with multiple periods (more than two), the standard DID approach assumes homogeneous treatment effects regardless of the duration of treatment exposure. It also cannot handle scenarios with more than two treatment groups with different policy start dates. To relax these inherent limitations, we used a heterogeneous difference-in-differences approach that extends DID by allowing for flexible treatment effects both across groups and time. For instance, consider two cohorts or units that receive the treatment at different times.^37–39^ This can be represented intuitively as:

3 $${Y}_{it}={\alpha}_{i}+{\lambda}_{t}+{\delta}_{1}{D}_{it{g}_{i1}}+{\delta}_2{D}_{it{g}_{i2}}+{X}_{it}^{\prime}\beta +{\epsilon}_{it}$$

where *g*_*i*1_ and *g*_*i*2_ indicate belonging to treatment groups 1 or 2 (in our case, state 1 or 2), respectively. The delta parameters (*δ*_1_ and *δ*_2_) represent the heterogeneous treatment effects for each group or unit. This equation can be further extended to allow for different effects over time. In our analysis, we adjusted state-level characteristics, including the unemployment rate, the percentage of state population with health insurance, the log of median household income, the log of population, mean age, percentage of the state population that is male, percentage of the state population that is white, blood alcohol concentration laws, state beer taxes (inflation-adjusted), and whether a state had Medicaid expansion in a particular year. We also conducted an event analysis to assess pre-existing differences and dynamic effects. Event analyses determine whether significant disparities exist in the outcome variable of interest between the treatment and control groups. In this context, differences observed prior to the policy implementation serve as a measure of any pre-existing differences that could be wrongly attributed to the policy (i.e., policy endogeneity). The analysis also explores how the effects of the policy fluctuate with the duration of exposure, providing insights into the dynamic effects of the policy.

## RESULTS

Table 1 presents the average treatment effects on the treated (ATET) from the heterogeneous difference-in-differences regression results for the two treatment cohorts, Colorado and Illinois, across various time periods, with alcohol as the primary substance treatment. The HDID findings indicate a significant relationship between the repeal of AELs and increased healthcare professionals’ treatment admissions referrals. In the year leading up to the policy change, there was a decline in treatment admissions. However, in the year following the policy’s implementation, we found an increase in admissions. An intriguing trend was particularly noticeable in Colorado, where, starting in 2009, there was a significant increase in treatment admissions. The higher number of admissions remained persistent through 2011. Specifically, the repeal of AELs was associated with an additional 929 treatment admissions (*p*-value = 0.078) when compared to states with no similar policy (the control group). Furthermore, in 2011, the repeal of AEL was associated with increased treatment admissions (ATET = 2183, *p*-value < 0.001) relative to the control group.

**Table 1:** Alcohol Exclusion Laws Prohibition and Treatment Admissions Referrals By Healthcare Professionals for Treatment States, 2003–2017

**Cohort**	**Year**	**ATET**	**95% CI**	***p*-value**
**Colorado**	2003	−4092.56	−4919.84, −3265.28	< 0.001
	2004	2237.96	−553.90, 5029.82	0.116
	2005	329.37	−1161.22, 1819.96	0.665
	2006	173.24	−844.70, 1191.18	0.739
	2007	−31.76	−408.66, 345.15	0.869
	2008	448.05	−49.53, 945.64	0.078
	2009	929.28	259.13, 1599.38	0.007
	2010	1006.76	−0.55, 2014.06	0.05
	2011	2182.89	1192.59, 3173.19	< 0.001
	2012	804.18	−352.41, 1960.56	0.173
	2013	−110.57	−1400.62, 1179.49	0.867
	2014	−900.87	−2386.17, 584.42	0.235
	2015	−2529.63	−4326.56, −732.71	0.006
	2016	−1769.55	−3927.35, 388.26	0.108
	2017	−1919.51	−5129.85, 1290.82	0.241
**Illinois**	2003	96.29	−803.32, 995.90	0.834
	2004	2479.17	−859.64, 5817.98	0.146
	2005	−215.52	−1236.87, 805.83	0.679
	2006	820.52	−515.84, 2156.87	0.229
	2007	−492.37	−808.96, −175.79	0.002
	2008	858.67	594.20, 1123.14	< 0.001
	2009	1392.02	605.81, 2178.24	0.001
	2010	1770.13	321.32, 3218.94	0.017
	2011	1856.61	458.53, 3254.69	0.009
	2012	966.38	−941.07, 2873.84	0.321
	2013	1302.39	−1198.78, 3803.57	0.307
	2014	1480.71	−1337.74, 4299.27	0.303
	2015	3111.57	−149.65, 6372.78	0.061
	2016	1955.39	−1780.21, 5690.98	0.305
	2017	3232.80	−1779.23, 8042.83	0.188

*ATET* average treatment effects on the treated. *CI* confidence interval. *p*-values are two-tailed

Turning our attention to Illinois, which repealed AEL in 2008, we see a similar trend. The policy change was significantly associated with higher treatment admissions referred by healthcare professionals from 2008 to 2011. There were more treatment admissions in 2008 (ATET = 859, *p*-value < 0.001), 2009 (ATET = 1392, *p*-value < 0.001), 2010 (ATET = 1770, *p*-value = 0.017), and 2011 (ATET = 1857, *p*-value = 0.009).

**Figure 1:**
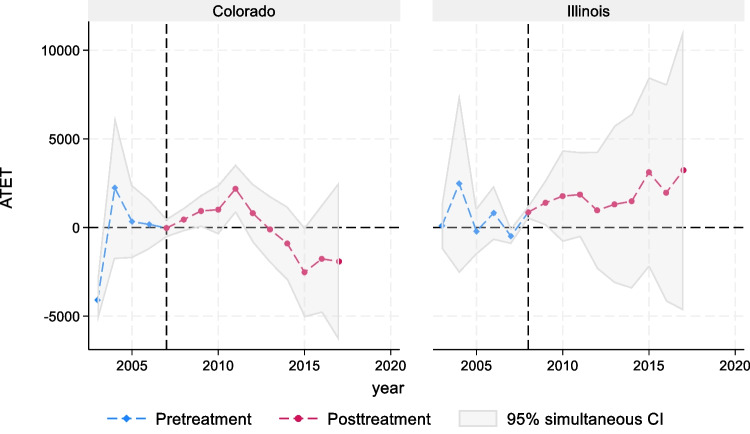
The effects of alcohol exclusion laws on treatment admissions referrals by healthcare professionals, 2003–2017.

Figure 1 illustrates the average treatment effects on the treated and their pointwise confidence intervals, both before and after the treatment. This is demonstrated separately for each treatment state, Colorado and Illinois. The figure effectively depicts the estimated policy effect parameter over time. Supplementary Table 1 and Figure 1 show the analysis results of the number of admissions referred by healthcare professionals for alcohol-related issues as the primary, secondary, or tertiary substance use disorder diagnosis. We found a consistent and significant increase in admissions in the immediate years after the repeal of AEL in both Colorado and Illinois from 2008 to 2011, relative to the control states.

**Figure 2:**
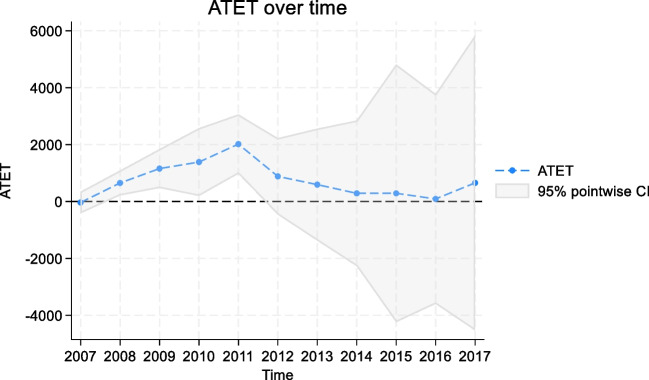
The aggregate effects of alcohol exclusion laws on treatment admissions referrals by healthcare professionals, 2007–2017.

The combined average treatment effect on the treated, which merges two treatment states, is presented in Table 2. AEL repeal in Colorado and Illinois was significantly associated with higher treatment admissions from 2008 to 2011 (2008 ATET = 653, *p*-value = 0.003, 2009 ATET = 1161, *p*-value = 0.001, 2010 ATET = 1388, *p*-value = 0.022, and 2011 ATET = 2020, *p*-value < 0.001). Figure 2 presents the visual display of the treatment effects for each one of the post-treatment periods. In Supplementary Table 2 and Figure 2, we found consistent results for alcohol-related treatment admissions (i.e., admissions with alcohol as the primary, secondary, or tertiary substance use disorder diagnosis).

**Table 2:** Alcohol Exclusion Laws and Treatment Admissions Referrals by Healthcare Professionals, Aggregate Over Time, 2007–2017

**Years**	**ATET**	**95% CI**	***p*-value**
2007	−31.76	−408.66, 345.15	0.869
2008	653.36	217.12, 1089.60	0.003
2009	1160.64	479.01, 1842.27	0.001
2010	1388.45	200.05, 2576.84	0.022
2011	2019.75	979.40, 3060.10	< 0.001
2012	885.23	−456.51, 2226.97	0.196
2013	595.91	−1368.05, 2559.88	0.552
2014	289.92	−2266.58, 2846.41	0.824
2015	290.97	−4235.39, 4817.32	0.900
2016	92.92	−3592.00, 3777.84	0.961
2017	656.64	−4526.44, 5839.73	0.804

*ATET* average treatment effects on the treated. *CI* confidence interval. *p*-values are two-tailed

**Figure 3:**
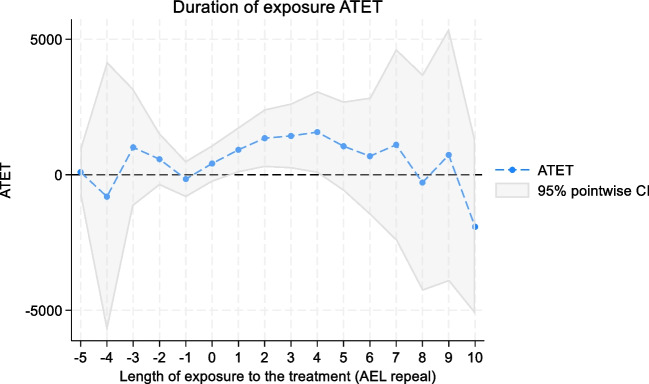
The effects of alcohol exclusion laws on treatment admissions referrals by healthcare professionals, duration of exposure, 2002–2017.

Table 3 presents the findings of an event analysis that examines pre-existing differences as well as dynamic effects between the treatment and control states. The results show that in the five periods leading up to the treatment onset, there is no significant effect on treatment admissions at any point during this pre-treatment exposure period. This suggests that prior to the policy change of the treatment, there were no notable changes in treatment admissions. At the point of treatment onset (denoted as 0), there is an increase in treatment admissions; however, this increase is not statistically significant. In the extended observation period post-treatment, we found that a longer duration of exposure to AEL repeal is associated with higher treatment admissions. This trend continues up to the fourth year post-treatment, and after this point, the effect appears to dissipate (Fig. 3). Supplementary Table 3 and Figure 3 show similar results using any alcohol-related treatment admissions as the outcome.

**Table 3:** Alcohol Exclusion Laws and Treatment Admissions Referrals by Healthcare Professionals, Duration of Exposure, 2002–2017

**Exposure**	**ATET**	**95% CI**	***p*-value**
−5	96.29	−803.32, 995.90	0.834
−4	−806.70	−5781.43, 4168.04	0.751
−3	1011.22	−1143.50, 3165.93	0.358
−2	574.95	−384.24, 1534.13	0.240
−1	−159.57	−824.14, 505.01	0.638
0	413.46	−258.32, 1085.23	0.228
1	920.04	90.71, 1749.36	0.030
2	1349.69	286.69, 2412.70	0.013
3	1431.68	237.65, 2625.72	0.019
4	1574.64	70.78, 3078.49	0.040
5	1053.24	−593.33, 2699.80	0.210
6	685.07	−1471.92, 2842.06	0.534
7	1105.35	−2422.26, 4632.95	0.539
8	−287.12	−4275.47, 3701.22	0.888
9	731.63	−3926.98, 5390.23	0.758
10	−1919.51	−5129.85, 1290.82	0.241

*ATET* average treatment effects on the treated. *CI* confidence interval. *p*-values are two-tailed

## DISCUSSION

This study investigated how the repeal of AELs in Colorado and Illinois was associated with the number of alcohol-related treatment admissions. AELs allow insurers to deny claims for injuries or illnesses caused by alcohol impairment. Twenty states have repealed AELs, with sixteen of those states also explicitly banning alcohol exclusion. There are variations in the scope and applicability of AEL prohibitions. For example, among the sixteen states that have explicitly banned alcohol exclusions, only Colorado and Illinois have also prohibited them in both health and accident insurance policies. Our study builds upon previous research that suggested an increase in treatment utilization following the repeal of AELs.^22^ We hypothesized a similar outcome for the unique form of AEL repeal adopted by Colorado and Illinois, and our findings confirmed this hypothesis. Specifically, we found a significant increase in treatment admissions in these states from 2008 to 2011, post-AEL repeal. Our event study further revealed that a longer duration of exposure to the AEL repeal was associated with higher treatment admissions. However, this effect seemed to diminish after the fourth year post-treatment. This suggests that the impact of AEL repeal on treatment admissions may dissipate over time.

Our findings can be interpreted through several potential explanations. Alcohol exclusion laws may discourage healthcare professionals from providing alcohol screening and intervention,^25,33,40^ which has been shown to reduce alcohol consumption and the risk of injury recurrence.^29^ In states that have adopted the Uniform Policy Provision Law (UPPL) with alcohol exclusions, unscreened patients may not receive treatment for potential alcohol-related disorders. This could lead to a higher likelihood of patients being referred to alcohol treatment in states that have repealed this deterrent. Colorado and Illinois, for instance, have not only repealed laws that permit these exclusions but have also uniquely prohibited such exclusions in both health and accident insurance. We observed an increase in treatment admissions referrals post-prohibition in both states, with a change in aggregate admissions per year between 2008 and 2011. This suggests that the distinct forms of alcohol exclusion repeal in Colorado and Illinois may have positively impacted alcohol treatment admissions referrals.

However, there were variations between the two states regarding the timing of change and the number of admissions by year. Unlike Colorado, the flow of treatment admissions in Illinois were immediately significant in the year of treatment (2008). Also, despite a significant aggregate change for the two states, increased admissions trends were not uniform over time. Although both Colorado and Illinois banned alcohol exclusions for health and accident insurance, they differed in both the scope and the wording of their repeal bills. The Colorado bill explicitly stated that insurers could not limit or exclude benefits if the insured or a covered dependent was injured while intoxicated.^41^ The Illinois bill also prohibited insurers from denying payments, but only if intoxication was the sole reason for the denial. This could explain observed differences between Colorado and Illinois in referrals where alcohol was the primary, secondary, or tertiary substance used. Additionally, the Illinois bill allowed insurers to apply deductibles, copayments, coinsurance, or annual or maximum payment limits to the coverage required by this section as long as they were consistent with other similar coverage under the plan.^42^ We draw attention to the above variations in the scope of AEL repeal and prohibition laws in these two states, highlighting the context to enhance the interpretation of our findings.

While this study provides valuable insights, it is important to note a few limitations. First, our analysis may not fully account for the potential confounding effects of local policies or other factors that changed in Colorado after the repeal of AELs. Second, our data from TEDS only includes treatment admissions at institutions receiving public funding. This excludes entirely private institutions, potentially limiting the generalizability of our findings. Third, our data set does not track individual patients across multiple treatment episodes. This could lead to double-counting of some cases, possibly overestimating the treatment demand. Fourth, we could not account for variations in quality and inclusion criteria for TEDS data collection across different states. Additionally, data related to general setting treatment (e.g., psychiatrist) might not have been included in TEDS, further complicating our analysis.

## CONCLUSIONS

This study examined the relationship between AEL repeal and alcohol-related treatment admissions in Colorado and Illinois, two states that had uniquely strong repeal laws. AELs allow insurers to deny coverage for injuries or illnesses caused by alcohol intoxication. Our study showed that strong AEL repeal was significantly associated with increased alcohol-related treatment admissions in these two states. However, our event study also revealed that the effect of the AEL repeals diminished over time. Nonetheless, our results suggest that banning alcohol exclusions in both health and accident insurance increased the treatment opportunities for alcohol-related disorders. States may benefit from considering the removal and prohibition of alcohol exclusions in health and accident insurance, as this could improve the treatment outcomes for alcohol-related disorders.

### Supplementary Information

ESM 1 (DOCX 89 kb)

## Declarations:

### Disclaimer:

The sponsors had no role in the design and conduct of the study, collection, management, analysis, and interpretation of the data; preparation, review, or approval of the manuscript; and decision to submit the manuscript for publication.
