# Supplementary material for: Treatment Referrals Post-prohibition of Alcohol Exclusion Laws: Evidence from Colorado and Illinois
Source: J Gen Intern Med. 2024 Jan 2;39(9):1649–56. doi: 10.1007/s11606-023-08544-2 (PMC11254860; doi:10.1007/s11606-023-08544-2)
Supplement: Supplementary file 1 — (DOCX 89 kb) [file 11606_2023_8544_MOESM1_ESM.docx]

**Table 1:** Alcohol Exclusion Laws prohibition and any alcohol-related treatment admissions referrals by healthcare professionals for treatment states, 2003-2017

| **Cohort** | **Year** | **ATET** | **95% CI** | **P-value** |
| --- | --- | --- | --- | --- |
| **Colorado** | **2003** | -4476.69 | -5245.99, -3707.40 | < .001 |
|  | **2004** | 2903.88 | -434.08, 6241.83 | 0.088 |
|  | **2005** | 606.43 | -1909.63, 3122.50 | 0.637 |
|  | **2006** | -16.18 | -1557.19, 1524.84 | 0.984 |
|  | **2007** | 25.95 | -516.01, 567.91 | 0.925 |
|  | **2008** | 779.7 | -9.72, 1569.12 | 0.053 |
|  | **2009** | 1409.68 | 214.64, 2604.71 | 0.021 |
|  | **2010** | 1741.34 | 122.63, 3360.05 | 0.035 |
|  | **2011** | 2632.66 | 983.69, 4281.64 | 0.002 |
|  | **2012** | 1311.44 | -614.53, 3237.41 | 0.182 |
|  | **2013** | 369.05 | -1840.35, 2578.46 | 0.743 |
|  | **2014** | -287.07 | -2853.03, 2278.89 | 0.826 |
|  | **2015** | -1950.05 | -4987.08, 1086.98 | 0.208 |
|  | **2016** | -1101.05 | -4673.98, 2471.87 | 0.546 |
|  | **2017** | -994.01 | -6414.02, 4425.99 | 0.719 |
|  |  |  |  |  |
| **Illinois** | **2003** | -374.60 | -1253.63, 504.43 | 0.404 |
|  | **2004** | 2565.36 | -1417.73, 6548.45 | 0.207 |
|  | **2005** | -201.05 | -1930.47, 1528.36 | 0.820 |
|  | **2006** | 1406.76 | -535.13, 3348.65 | 0.156 |
|  | **2007** | -285.81 | -670.90, 99.27 | 0.146 |
|  | **2008** | 1504.24 | 757.98, 2250.51 | < .001 |
|  | **2009** | 2153.29 | 518.28, 3788.31 | 0.010 |
|  | **2010** | 2507.52 | 148.53, 4866.52 | 0.037 |
|  | **2011** | 2792.48 | 427.11, 5157.86 | 0.021 |
|  | **2012** | 1090.42 | -2032.00, 4212.84 | 0.494 |
|  | **2013** | 1523.44 | -2559.57, 5606.45 | 0.465 |
|  | **2014** | 1827.62 | -2789.20, 6444.44 | 0.438 |
|  | **2015** | 4486.52 | -837.18, 9810.22 | 0.099 |
|  | **2016** | 2645.62 | -3418.83, 8710.08 | 0.393 |
|  | **2017** | 4924.28 | -2946.93, 12795.50 | 0.220 |

ATET average treatment effects on the treated. CI denotes confidence interval. P-values are two-tailed.

**Table 2:** Alcohol Exclusion Laws and any alcohol-related treatment admissions referrals by healthcare professionals, aggregate over time, 2007-2017

| **Time** | **ATET** | **95% CI** | **P-value** |
| --- | --- | --- | --- |
| **2007** | 25.95169 | -516.01, 567.91 | 0.925 |
| **2008** | 1141.974 | 316.19, 1967.76 | 0.007 |
| **2009** | 1781.485 | 466.30, 3096.67 | 0.008 |
| **2010** | 2124.433 | 294.97, 3953.90 | 0.023 |
| **2011** | 2712.573 | 961.14, 4464.00 | 0.002 |
| **2012** | 1200.929 | -1034.23, 3436.09 | 0.292 |
| **2013** | 946.2481 | -1999.42, 3891.92 | 0.529 |
| **2014** | 770.2758 | -2802.36, 4342.91 | 0.673 |
| **2015** | 1268.233 | -4578.09, 7114.56 | 0.671 |
| **2016** | 772.2837 | -4271.37, 5815.94 | 0.764 |
| **2017** | 1965.134 | -5510.52, 9440.79 | 0.606 |

ATET average treatment effects on the treated. CI denotes confidence interval. P-values are two-tailed.

**Table 3:** Alcohol Exclusion Laws and any alcohol-related treatment admissions referrals by healthcare professionals, duration of exposure, 2002-2017

| **Exposure** | **ATET** | **95% CI** | **P-value** |
| --- | --- | --- | --- |
| **-5** | -374.60 | -1253.63, 504.43 | 0.404 |
| **-4** | -955.67 | -6336.47, 4425.13 | 0.728 |
| **-3** | 1351.41 | -1315.45, 4018.27 | 0.321 |
| **-2** | 1006.60 | -530.54, 2543.74 | 0.199 |
| **-1** | -150.99 | -925.81, 623.82 | 0.702 |
| **0** | 765.10 | -378.20, 1908.39 | 0.190 |
| **1** | 1466.50 | 53.61, 2879.39 | 0.042 |
| **2** | 1958.60 | 243.77, 3673.44 | 0.025 |
| **3** | 2266.91 | 366.80, 4167.03 | 0.019 |
| **4** | 1861.54 | -485.85, 4208.94 | 0.120 |
| **5** | 1417.44 | -1260.35, 4095.23 | 0.300 |
| **6** | 1098.34 | -2153.19, 4349.87 | 0.508 |
| **7** | 2099.72 | -2791.44, 6990.89 | 0.400 |
| **8** | 347.78 | -4858.22, 5553.79 | 0.896 |
| **9** | 1911.61 | -4709.44, 8532.67 | 0.571 |
| **10** | -994.01 | -6414.02, 4425.99 | 0.719 |

ATET average treatment effects on the treated. CI denotes confidence interval. P-values are two-tailed.

**Figure 1:** The effects of Alcohol Exclusion Laws on alcohol-related treatment admissions referrals by healthcare professionals, 2003-2017

**Figure 2:** The aggregate effects of Alcohol Exclusion Laws on alcohol-related treatment admissions referrals by healthcare professionals, 2007-2017

**Figure 3:** The effects of Alcohol Exclusion Laws on alcohol-related treatment admissions referrals by healthcare professionals, duration of exposure, 2002-2017
